# Supplementary material for: Optimizing strategies for slowing the spread of invasive species
Source: PLoS Comput Biol. 2024 Apr 3;20(4):e1011996. doi: 10.1371/journal.pcbi.1011996 (PMC11018280; doi:10.1371/journal.pcbi.1011996)
Supplement: Attachment — Submitted filename: Lampert_Response_Reviewers.pdf [file pcbi.1011996.s001.pdf]

Reviewer 1

I sincerely thank the Reviewer for carefully reading the revised version of the manuscript and for the helpful comments. Below please find the Reviewer's comment (black fonts), followed by our response (blue & bold fonts). The submission includes two versions of the revised manuscript – one without marked changes and one in which changes from the previous version are highlighted in yellow.

This paper presents an algorithm that is capable of identifying the efficient control of an invader in an general spatial-dynamic model. The species spreads via a dispersal kernal. The marginal efficiency of treatment depends on the adult population density which varies over space. As such the efficient solution is not a uniform level of control at all points in space. Costs savings can be generated by applying varying levels of control at different points in space. The optimal control identifies where and when to apply control to achieve a target rate of propagation.

I enjoyed reading this paper. It is a nice example of using computational tools to address a growing biological problem. I also don't see anything fundamentally wrong with the algorithm itself. However, I do have three general concerns about the way the model results are framed and presented to readers followed by several specific question/suggestions.

I thank the Reviewer for the positive view of the paper and for the instructive three comments.

1. I would like to see the model applied to a real-world case study. While the author does apply the algorithm to a generic example, I think an application to a specific invasive species will be beneficial for two reasons. First, it will provide an indication of the magnitude of cost savings achieved with the algorithm. Yes there are theoretical cost savings that can be achieved with the algorithm, but it would help to indicate to the reader how large these cost savings may be. This is exactly the type of exercise that an algorithmic approach to invasive species control should provide over dynamic programming of Pontryagin's maximum principle. The second reason for the case study is it will give the reader an indication of the data required to believably apply the algorithm. Reliable data on the density of invasive

species at different points in space is notoriously difficult to obtain. The author should convince the reader that the data needed to benefit from the algorithm exists.

The Reviewer suggests applying the algorithm to a real-world case study. In response, I developed and analyzed a model for the spongy moth (*Lymantria dispar*) population in North America. For decades, efforts have been underway to slow the moth's spread from the North-Eastern US. One of the main tools for slowing the spread has been mating disruption – using pheromones that prevent males from locating the females. To characterize the moth's population dynamics, I constructed a model that includes discrete-time dynamics (characterizing the moth's annual cycle) and a birth function that incorporates the effect of mating disruption on the number of offspring. This model is described in detail in a new sub-section, "Functional forms – spongy moth population model" in lines 183-229. The forms of the functions are based on spongy moth dynamics (lines 183-213), with parameter value ranges based on the literature (lines 214-229).

The revised manuscript presents the results for both the spongy moth model and the general model analyzed in the previous version of the manuscript. Both models were analyzed using the same general algorithm. Figure 3 is a new figure that shows that the algorithm converges to the same solution for the spongy moth model from different initial conditions (where Fig. 2 shows a similar result for the general model). The algorithm significantly improves optimal treatment strategies over non-optimal ones for the spongy moth model: "slowing the front but keeping its shape similar to that of an untreated population would result in an annual cost of treatment (ACT) that is higher by tens or hundreds of percentages than the ACT of the optimal treatment that slows the population's spread to the same speed. This result appears in the analysis of the general model (compare Figs. 2A where $\mathrm{ACT} = 28.58$ and Fig. 2D where $\mathrm{ACT} = 8.58$) as well as in the analysis of the spongy moth case study (compare Figs. 3A in which $\mathrm{ACT} = \$278.2K$ and Fig. 3D in which $\mathrm{ACT} = \$56.29\$$, where ACT is measured in units of thousands of USD per one-kilometer strip of land over which the front propagates). A similar improvement in cost-efficiency is obtained if one uses the optimal treatment instead of a treatment that maintains a front with a shape of a straight line. This efficiency gain is evident in both the general model (compare Figs. 2E where $\mathrm{ACT} = 25.82$ and Fig. 2H where $\mathrm{ACT} = 8.52$) as

and the spongy moth case study (compare Figs. 3A where $\text{ACT} = \$197.3K$ and Fig. 3D where $\text{ACT} = \$56.15K$)" (lines 412-423).

In turn, Fig. 5 is another new figure showing how the annual cost of treatment of the optimal solution (ACT^*) depends on v and on other population parameters in the spongy moth model. This analysis enables us to use empirical estimates of the benefits of slowing the spread to identify the optimal v for various scenarios: "In particular, (Leuschner et al. 1996) have conducted a thorough study to valuate the damage incurred by the spongy moth. They found that slowing the spread by one kilometer annually across a one-kilometer strip yields a benefit of 16K USD per year… Our results show that, for parameter values used in Fig. 5, the optimal target speed is $v = -2.4$ km/year if $k\lambda_0 = 100$; $v = 4.8$ km/year if $k\lambda_0 = 250$; and $v = 9.6$ km/year if $k\lambda_0 = 1000$" (lines 501-509).

2. More effort needs to be devoted to convincing readers that the algorithm provides a good approximation of the actual solution. One approach would be to demonstrate how well the algorithm approximates the closed form solution presented in the paper. It is important to present both the global error as well as showing how the error varies over space. My guess is that the approximation error gets worse near the population front due to the nonlinearities.

The Reviewer asks that we explain how the algorithm's outputs, A^{opt}, differ from closed-form solutions of the optimal treatment. However, note that I do not have any closed-form solution of the optimal solution. The only closed-form solutions (Eqs. (14, 16)) are for the local value of A that reduces the population from a higher density ($n_1(x)$) to a lower one ($\tilde{n}(x + \Delta x)$). The complete spatial-temporal model is way too complicated to solve analytically due to the nature of dynamics with an integral kernel. Note that most previous studies have focused on much simpler models with diffusion, and even then, there were no closed-form solutions in most cases.

Nonetheless, what is apparent from the solutions is that the algorithm consistently converges to the same optimal shape, regardless of the initial front shape (e.g., Figs. 2 and 3). The revised manuscript's additional analysis with the spongy moth model in Fig. 3 confirms the algorithm's convergence to a consistent solution across various initial shapes in both models. Additionally, I tested other kinds of modifications to the front shape, none of which improved upon the algorithm's optimal solution. (I do not describe these attempts

in the manuscript because, as I mentioned, they did not provide any improvement to the algorithm that is described in the manuscript.) This at least strengthens the hypothesis that the solutions found by the algorithm's solutions are optimal or near-optimal for the cases examined.

3. Can the model accommodate species that spread via human-mediated, long-distance dispersal where populations establish well in advance of the primary invasion front? This would seem to allow for more complex functions for n(x,t) that may give the algorithm trouble. Or are you assuming that delta_t is long enough to eliminate any concerns about isolated populations?

The Reviewer inquires whether the current algorithm is suitable for cases where human-mediated dispersal leads to isolated populations that are very distant from the primary invasion front. The answer is that the present algorithm allows for long-distance dispersal via suitable choices of the dispersal kernel, but it does assume that the shape of the front is monotone. Investigating non-monotone front shapes with stochastic dynamics that could lead to isolated locations with higher densities is a possible future direction. In the revised manuscript, I clarify that "the dynamics in our model are deterministic, whereas real-world invasions exhibit stochastic dynamics and uncertainty. In particular, invasion could appear in more distant locations, and the locations where new populations appear might not be predictable. Future studies could address this issue by examining how to combine suppression with surveillance that exposes the distribution of the invader and detects areas to which it spreads faster (Hauser and McCarthy 2009, Epanchin-Niell et al. 2012)" (lines 526-531).

I sincerely thank the Reviewer again for the valuable comments that helped to improve the manuscript.

Reviewer 2

I sincerely thank the Reviewer for carefully reading the revised version of the manuscript and for the helpful comments. Below please find the Reviewer's comment (black fonts), followed by our response (blue & bold fonts). The submission includes two versions of the revised manuscript – one without marked changes and one in which changes from the previous version are highlighted in yellow.

This is a well-crafted and useful paper on optimal containment of a biosecurity event. I would only encourage more care with the existing literature so that readers can properly judge the value of the paper. For example:

1. "Few bioeconomic studies have explored the optimal containment of an invasive species to stop or slow its spread." See:

https://www.sciencedirect.com/science/article/pii/S0921800911003855
and
https://onlinelibrary.wiley.com/doi/abs/10.1111/1467-8489.12305
and
https://esajournals.onlinelibrary.wiley.com/doi/abs/10.1002/eap.2319
to name a few

I thank the Reviewer for finding the paper well-crafted and useful. I also thank the Reviewer for suggesting these three relevant papers. I added these citations to the manuscript and I write that "Biosecurity measures often combine prevention, detection, and early eradication to prevent the establishment of invasive species (Rout et al. 2011, Kompas et al. 2019, Welsh et al. 2021)" (lines 31-33).

This adds to the numerous citations that are already in the manuscript to studies that are tailored more specifically to finding the optimal spatial allocation of treatments to slow the species' spread in lines 66-75. In particular, in line 66, I changed "few bioeconomic studies…" to "several bioeconomic studies…", which gives a more accurate impression for the numerous studies cited in that paragraph.

2. "One reason for the scarcity of studies that explored optimal approaches to slowing populations' spread is that standard optimization algorithms are limited in their capacity to solve spatially-extended dynamical optimization problems over large areas." Check, for example:

https://esajournals.onlinelibrary.wiley.com/doi/abs/10.1002/eap.2449

The Reviewer mentions another relevant paper. I added this citation and another one that "examined the optimal strategies for the surveillance of small populations of the species (Cacho et al. 2010, Epanchin-Niell et al. 2012, Nguyen et al. 2021)" (lines 68-70).

Otherwise the method is valuable and worth publishing in some form. The algorithm for finding an optimal treatment strategy is certainly a nice innovation, with clear results.

I thank the Reviewer for acknowledging that the algorithm is certainly a nice innovation with clear results.

3. Also, I would only hope for some additional and *practical* application of the method and algorithm -- a case study that can illustrate the framework and its practical application. It is a big ask, but I think necessary and I can think of a number of potential case studies already (e.g., the attempt to eradicate and contain RIFA in Australia; the eradication of Papaya Fruit Fly in Queensland, many in North America, etc).

The Reviewer suggests to connect the algorithm to a real-world case study – a similar suggestion to that of the other Reviewer. In response, I developed and analyzed a model for the spongy moth (*Lymantria dispar*) population in North America. For decades, efforts have been underway to slow the moth's spread from the North-Eastern US. One of the main tools for slowing the spread has been mating disruption – using pheromones that prevent males from locating the females. To characterize the moth's population dynamics, I constructed a model that includes discrete-time dynamics (characterizing the moth's annual cycle) and a birth function that incorporates the effect of mating disruption on the number of offspring. This model is described in detail in a new sub-section, "Functional forms – spongy moth population model" in lines 183-229. The forms of the functions are based on spongy moth dynamics (lines 183-213), with parameter value ranges based on the literature (lines 214-229).

The revised manuscript presents the results for both the spongy moth model and the general model analyzed in the previous version of the manuscript. Both models were analyzed using the same algorithm. Figure 3 is a new figure that shows that the algorithm converges to the same solution for the spongy moth model from different initial conditions (where Fig. 2 shows a similar result for the general model). As in the general model, also in the spongy moth model, the algorithm significantly improves optimal treatment strategies over non-optimal ones, as shown in Fig. 3 and explained in lines 412-423. In turn, Fig. 5 is another new figure showing how the annual cost of treatment of the optimal solution (ACT^*) depends on v and on other population parameters in the spongy moth model. This analysis enables us to use empirical estimates from the literature of the benefits of slowing the spread to identify the optimal v for various scenarios (e.g., lines 501-509).

I sincerely thank the Reviewer again for the valuable comments that helped to improve the manuscript.
